# Supplementary material for: Standardization of Molecular MRD Levels in AML Using an Integral Vector Bearing ABL and the Mutation of Interest
Source: Cancers (Basel). 2023 Nov 10;15(22):5360. doi: 10.3390/cancers15225360 (PMC10670136; doi:10.3390/cancers15225360)
Supplement: Supplementary file 1 [file cancers-15-05360-s001.zip › cancers-2684400-supplementary.pdf]

Table S1 Mutations, primers and probes of presented mutations

Patient #	Mutated gene	Mutation	Mutation specific Primer	Second Primer	Probe
1	RUNX1	NM_001754.4 c.820_820delC	5'- ACCACGGTGGGGATGGTaGA -3	5'- TTCCGAGCGGCTCAGTGAAC -3'	6-Fam--CTGCGGCGCACAGCCATGAGGGTC-BHQ1
2	RUNX1	NM_001754.4 c.527C>T p.Thr176Ile	5'- GTG GGT TTG TGA AGA CAG TGA aGA -3'	5'-TGATGGCTGGCAATGATGAAAAC -3'	6-Fam--GCTGAGCTGAGAAATGCTAC-BHQ1
3	RUNX1	NM_001754.4 del c.706_718delMet236HisfsTer14	5'- CTGCGGCGCACAGCCCA -3'	5′-CC AGG TAT TGG TAG GAC TGA TCG-3′	6-Fam--CCACGCCCAACCCTCGTGCCTCCCTG-BHQ1
4	RUNX1	NM_001754. 4c.507_508dup p.Gly170GlufsTer7, 513A>T p.Lys171Asn	5′-TCAGAGTGAAGCTATTCCCTCTC-3′	5'- CAC TGT GAT GGC TGG CAA TG -3'	6-Fam--GCTGAGCTGAGAAATGCTAC-MBHQ1
5	RUNX1	c.694C>T p.Arg232Trp	5'- CTC AGT GAA CTG GAG CAG CTa T -3'	5'- TT GTA TCC TGC ATC TGA CTC TG -3'	6-Fam-CCACGCCCAACCCTCGTGCCTCCCTG-BHQ1
6	RUNX1	NM_001754.4:c.720_733del p.His242AlafsTer14	5'- GCG TGG G-GG GCT GAC CCT -3'	5'- ACC ATC ACT GTC TTC ACA AAC C -3'	6-Fam- CCGCAAGTCGCCACCTACCA-BHQ1
7	RUNX1	NM_001754.4:c.663delinsGGA p.Phe221LeufsTer17	5'-TCCACTTCGACCGACAAACaC -3'	5'- GGA TGG TTG GAT CTG CCT TG -3'	6-FAM-CTGCGGCGCACAGCCATGAGGGTC-BHQ1
8	RUNX1	NM_001754.4 c.1029_1030insTC	5'- CGC GCT GCC CTC CAT CTC TC -3'	5'- GCGAGCTGGCTTGGAACGG -3'	6-Fam-CATCGGCATCGGCATGTCGGCCATG-BHQ1
9	NPM1	NM_002520.7(NPM1):c.863 ins ACGC (p.Trp288fs)	5'- AGGCTATTCAAGATCTCTACGC -3'	5'- CACGGTAGGGAAAGTTCTCAC -3'	6-Fam-GTAACAGTTGATATCTGGCTGTCC –BHQ1
10	NPM1	NM_002520.7(NPM1):c868insAAAA(p.Trp290fs)	5'-ctattcaagatctctggcagAAAAt-3'	5'-CACGGTAGGGAAAGTTCTCAC-3'	6-Fam--GTAACAGTTGATATCTGGCTGTCC –BHQ1
11	NPM1	NM_002520.7(NPM1):c.863insCATG(p.W288fs)	5'-GCTATTCAAGATCTCTGCATGG-3'	5'- CACGGTAGGGAAAGTTCTCAC -3'	6-FAM-GTAACAGTTGATATCTGGCTGTCC- BHQ1
12	NPM1	NM_002520.7(NPM1):c.863_864insTAAG	5′-gctattcaagatctctgTAAGg-3′	5'- CACGGTAGGGAAAGTTCTCAC -3'	6-FAM-GTAACAGTTGATATCTGGCTGTCC- BHQ1
13	NPM1	NM_002520.7(NPM1):c. 863insTAGG (p.Trp288fs)	5'- gctattcaa gatctctgtaggg-3'	5'- CACGGTAGGGAAAGTTCTCAC -3'	6-FAM-GTAACAGTTGATATCTGGCTGTCC- BHQ1
14	IDH2	NM_002168.3:c.419G>A R140Q	5'-GAAAAGTCCCAATGGAACTATCCrAGAACC/3SpC3/ -3'	5'-AGTCTGTGGCCTTGTACTG-3'	56-FAM/AAACATCCC/ZEN/ACGCCTAGTCCCTG/3IABkFQ
15	IDH2	NM_002168.3:c.419G>A R140Q	5'-GAAAAGTCCCAATGGAACTATCCrAGAACC/3SpC3/ -3'	5'-AGTCTGTGGCCTTGTACTG-3'	56-FAM/AAACATCCC/ZEN/ACGCCTAGTCCCTG/3IABkFQ
16	IDH2	NM_002168.3:c.515G>A R172K	5'-GCCCATCACCATTGGCArAGCACC/3SpC3-3'	5'-CTTGACACCACTGCCATCTT-3'	56-FAM/CGACCAGTA/ZEN/CAAGGCCACAGACTTT/3IABkFQ
17	IDH2	NM_002168.3:c.419G>A R140Q	5'-GAAAAGTCCCAATGGAACTATCCrAGAACC/3SpC3/ -3'	5'-AGTCTGTGGCCTTGTACTG-3'	56-FAM/AAACATCCC/ZEN/ACGCCTAGTCCCTG/3IABkFQ

18	IDH2	NM_002168.3:c.515G>A R172K	5'-GCCCATCACCATTGGCArAGCACC/3SpC3-3'	5'-CTTGACACCACTGCCATCTT-3'	56-FAM/CGACCAGTA/ZEN/CAAGGCCACAGACTTT/3IABkFQ
19	IDH1	NM_005896.3:c.394C>T R132C	5'-TGGGTAAAACCTATCATCATAGATT-3'	5'-AGTTATGTACCAGGTATGTCAC-3'	6-Fam-CCTACACACCAAGTGACGGAACCC-BHQ1
